# Congress report: Online workshop on assessment of technical files for blood screening in vitro diagnostics for sub‐Sahara African countries

**DOI:** 10.1111/tme.12925

**Published:** 2022-10-20

**Authors:** Herbert A. Mbunkah, Chancelar Kafere, Washington Samukange, Micha Nübling, Jens Reinhardt

**Affiliations:** ^1^ Division G3 – International Coordination, Regulatory Service Paul‐Ehrlich‐Institut Langen Germany; ^2^ Department of Biological Sciences Faculty of Science, University of Bamenda Bambili Cameroon; ^3^ Division of Haematology and Transfusion Medicine Paul‐Ehrlich‐Institut Langen Germany

**Keywords:** blood safety, blood transfusion, in vitro diagnostics, online workshop, technical requirements

## Abstract

**Objectives:**

The online workshop on IVD regulation was performed to broaden the understanding of the technical documentation needed for IVD licensing and the strategies to asses it.

**Background:**

Testing of blood donors and donations significantly reduces the risk of transmitting transfusion‐transmissible infections. Many test systems are commercially available, but not all meet the recommended sensitivity and specificity standards. Many African countries either lack functional structures for the regulation of IVDs this poses a threat to the quality of the blood supply.

**Materialsand Methods:**

The Paul‐Ehrlich‐Institut BloodTrain organised an online workshop in September 2021 to introduce staff from several National Regulatory Authorities (NRAs) in Africa to the regulation of IVD and the technical information that need to be provided by the manufacturers of blood screening IVD. Their evaluation was trained in practical exercises.

**Results:**

This online workshop brought together over hundred participants from NRAs of 12 African countries. Speakers from PEI, Blood Train, WHO and academia, with experience in IVD regulation trained participants in the various topics addressed during this workshop.

**Conclusions:**

This workshop presented a great starting point for most participating NRAs to set up and/or strengthen their regulatory structures for IVDs.

## 1 INTRODUCTION

Suitable testing of all blood donations is critical for the provision of safe blood for transfusion. Screening donated blood for transfusion‐transmissible infections (TTIs) such as HIV, HBV, HCV and syphilis significantly reduces the risk of transmission of these infections to recipients to very low levels. Similarly, blood group testing such as ABO and Rh typing and screening for red cell antibodies ensures transfusion of compatible blood, hence improves safety. Different systems, techniques and equipment are available for both TTI testing and blood group serology ranging from manual, semi‐automated to fully automated. To guarantee a safe and quality supply of blood and blood products in any country, there is need for thorough regulation of these testing systems by National Regulatory Authorities (NRAs). In the European Union (EU) for instance, in vitro diagnostics (IVDs) like those for blood screening belonging to higher risk categories undergo a conformity assessment procedure by a Notified Body that audits the quality management system and assesses the technical documentation of the device.^1^ Other requirements are confirmed during performance evaluation of the device. Only then can a European certificate (CE mark) be issued to confirm compliance with the EU requirements. The WHO though not a regulatory body, assists her Member States with prequalification assessments for medicinal products including IVDs.^1^

The Global Health Protection Programme (GHPP) was launched by the German Ministry of Health to contribute to international initiatives to strengthen infrastructure of health systems in low and middle income countries.^2^ One of the projects of the GHPP being implemented by the Paul‐Ehrlich‐Institut (PEI) is the BloodTrain, which is aimed at ensuring availability, safety and quality of blood and blood products in Africa, through strengthening of capacities of national regulatory systems for blood.^3^ Regulatory oversight of associated substances and medical devices including IVDs used in blood product preparation processes is one of the key regulatory functions for which capacity building is essential.

Although many test systems for the detection of infectious diseases are also commercially available in Africa, not all meet the recommended sensitivity and specificity standards for the reliable detection of TTIs. By virtue of them having a significant impact on the safety, quality and efficacy of blood and blood products, associated substances and medical devices including IVDs need to be thoroughly regulated. In 2012 a survey of East African Community partner states found that regulation of IVDs was a neglected area among member states.^4^ In most of the states, a legal framework for regulating medical products was present. However, their capacity to regulate IVDs was limited. A benchmarking exercise conducted by the PEI BloodTrain in 2018^5^ revealed that only three out of ten benchmarked NRAs in Africa have established functional structures for the regulation of medical devices and IVDs. In a 2020 survey (unpublished data) involving NRAs in Africa, conducted by the African Medical Devices Forum, only approximately 30% of participating NRAs had implemented basic level controls for the regulation of IVDs as prescribed in the WHO Global Model Regulatory Framework for Medical Devices including IVD medical devices.^6^

An IVD technical file embodies all the information that is held by a manufacturer in relation to a particular IVD product. These are detailed under the different subheadings of the technical file (see Box 1). The documentation is normally an output of the manufacturer's quality management system, and includes information generated throughout the design, development, production and monitoring phases of the IVD device.^7^ Conformity to the essential principles of safety and performance for an IVD device is often demonstrated in various sections of the technical dossier. Assessment of a technical file for an IVD device is one of the crucial regulatory functions that every sound medical device regulatory body must perform before approving the device for use in the country. Consequently, this workshop presented a starting point for the participating NRAs to set up and/or strengthen their regulatory structures for IVD assessment and licensing. The workshop was designed to address all subheadings of the technical file to give the participants a good introduction of the content of the dossier that will need to be assessed.

BOX 1 **Content of the technical documentation for in vitro diagnostics/medical devices**

The technical documentation for IVDs/MDs

- General and administrative information
- Device description
- Design and manufacturing information
- Quality management system
- Commercial and regulatory history
- Essential principles of safety and performance, and evidence of conformity
- Risk management
- Product performance evaluation: analytical and diagnostic studies
- Stability and robustness
- Labels and instructions for use
- Planned post‐marketing surveillance

Due to the Covid‐19 pandemic, this meeting had to be convened as a virtual training. This allowed the BloodTrain increase the number of participants to also include those from additional African countries that are presently not partner countries, where the BloodTrain activities usually focus.

## 2 THE WORKSHOP AND PARTICIPANTS

This online workshop organised by the PEI‐GHPP BloodTrain from 27–30 September 2021 brought together over a hundred participants—mostly regulators of medical devices and IVDs from NRAs of 12 African countries. These countries included Ethiopia, Ghana, Kenya, Liberia, Malawi, Nigeria, Rwanda, South Africa, Tanzania, Uganda, Zambia and Zimbabwe. The exact number cannot be evaluated, since some participants did not log onto the system and some used the system together with only one log‐in. Five of these countries (Ghana, Nigeria, Tanzania, Zambia and Zimbabwe) are BloodTrain partner countries while the rest are countries where benchmarking of blood regulatory systems was previously conducted by the BloodTrain. A variety of speakers from PEI, BloodTrain, WHO and academia, with experience in IVD regulatory matters were invited to school participants in the various topics addressed during this 4‐day workshop (see Appendix for the programme).

## 3 WORKSHOP OBJECTIVES AND PROGRAMME

The overall objective of this workshop was to establish and strengthen competencies in reviewing and assessing blood screening IVD technical files in the participating countries' NRAs. Specifically, we sought to:

- Familiarise participants with the fundamental principles of regulating IVDs,
- Develop and improve skills and competences in IVD technical file assessment,
- Share best practices in the regulation of blood screening IVDs,
- Discuss reliance as a practice in the regulation of IVDs with related existing platforms and systems for implementation,
- Familiarise participants with emergency use authorization/approval procedures for diagnostics.

Since the workshop was held virtually, it was decided to limit each day to a maximum of 3 h. From earlier experience, longer virtual meetings are less effective, as it is challenging for the participants to stay focused. The 4 days were thematically divided into a general part, where the role of IVDs and the elements of their successful regulation were presented (day 1), a part in which the technical file and its assessment was discussed in detail (days 2 and 3), and a last part in which experts from WHO explained their approaches to support the global availability of IVDs (day 4).

### 3.1 Day 1—Overview of regulation for blood screening IVDs

The workshop started with a brief welcome and introductory note from Dr. Jens Reinhardt (deputy project lead of the BloodTrain) on the background and mission of the BloodTrain and the objectives of the workshop. Dr. Micha Nübling from PEI proceeded with a detailed overview of the state‐of‐the‐art of IVD and their role of diagnostics in the blood supply. He pointed out that the prevention of infections as a hallmark of blood safety is achieved by multiple strategies, including the physical donor examination, the donor questionnaire and the testing of each donation for markers of infection. He also presented the history of blood testing and the correlated evolution of molecular testing methods. Starting in the 1970s, where the liver marker ALAT was used to exclude donors with a high probability of hepatitis B and C, to nowadays available multiplex PCR testing. This talk also brought to light the different levels of sensitivity of test systems and addressed the aspect, which level of sensitivity can be accepted by regulators. Dr Nübling also discussed regional differences in virus genotypes, which is also relevant for the selectivity of the assay and needs to be taken into account when deciding about the utility of an IVD kit in a specific area. By pointing towards the guidance documents and standard preparations that WHO provides, he concluded his presentation.

Ms. Agnes Kijo from WHO gave the next talk on the Elements of Regulatory Systems for Diagnostics based on the WHO Global Model Regulatory Framework for medical devices including IVDs. This framework document was published in 2017 and provides strategies to establish a regulatory control system starting from a basic level and expanding it. This talk created awareness on the basic level controls from pre‐market to post‐market, which countries in the early stages of regulating IVDs must strive to enforce. Enabling conditions for effective regulation of medical devices and IVDs were also discussed.

### 3.2 A snapshot of IVD regulation in different African countries

An overview of the IVD regulatory situation in four African countries was presented by Ms. Jeniva Rugaiza from TMDA, Mr. Frank Laban from ZAMRA, Dr. Edwin Nkansah from the Ghana FDA and Mr. Aina Olugbenga Stephen from NAFDAC. These NRAs have enforced some of the basic level regulatory controls from pre‐market to post‐market as recommended in the WHO Global Model Regulatory Framework for Medical Devices including IVDs. In all countries, the responsibility to control IVDs is defined by law and the NRAs are the responsible organisation tasked with this duty. The speakers presented the recent developments and the current challenges within the IVD regulation.

The day ended with a polling exercise led by Mr. Washington Samukange to gather feedback from participants. This exercise started with an introduction into the concept of WHO Global Benchmarking Tool Plus (GBT+) Blood, which allows to perform a globally standardised self‐assessment of NRAs and to identify gaps in their capability to stringently regulate medicines including blood and blood products. While at that time, medical device regulation was not implemented into the GBT+ Blood, the IVDs for blood screening are already in the focus of the tool. Subsequently, an anonymous online WebEx poll for all participants to indicate their level of implementation of IVD regulatory controls in their countries was performed.

### 3.3 Day 2—Assessment of the IVD technical file: Part 1

The second and the third day covered most of the subheadings of the technical file (see Box). Day 2 focused on a detailed understanding of the content of the technical file and approaches to the evaluation of the content. After a summary talk by Dr. Herbert Mbunkah (BloodTrain) about the composition of a typical IVD dossier, shortly summarising all subheadings, a more detailed insight into the subheadings were provided in the following talks. First Prof. Willy Urassa, an independent IVD consultant, trained participants on the *Essential Principles of Safety and Performance* for medical devices and IVDs. The presentation pointed to the conformity assessment that has to be provided by the manufacturer to prove to regulators that the IVD (or any other medical device) is safe and performs as intended. This conformity assessment uses systematic examination of the IVD to generate scientific evidence of the performance, for example the sensitivity and selectivity of the assay, the stability of the components and the robustness in the hand of the end user. It was further explained, how the risk management approach should be described in the technical file. This presentation helped to understand what kind of information need to be assessed and also pointed to some international standards for it, for example, those from the International Organisation for Standardisation (ISO) and the International Medical Devise Regulators Forum.

Dr. Mbunkah then discussed the *Design and Manufacturing Information* for an IVD that is required for assessment by the regulator. The design and/or manufacture includes all aspects from specification development, production, fabrication, assembly, processing, packaging, repackaging, labelling, relabelling, sterilisation, installation or remanufacturing of a medical/IVD device.^8^ All the information must comply with the aforementioned Essential Principles of Safety and Performance on one hand, and with the current Good Manufacturing Practices and the Quality System Regulation on the other. The manufacturer of the IVD needs to provide sufficient data on all these aspects. The presentation gave a detailed view on which information needs to be included under the different headers. The way to assess this information by the NRA was exemplified by the relevant parts of the checklist developed and routinely used by the WHO‐Prequalification Team, as discussed by Dr. Mbunkah. Dr. Joey Gouws (WHO Prequalification Programme) presented a holistic approach to addressing pertinent issues pertaining to the assessment of the *Quality Management System* for IVD products. This talk also highlighted the work of inspectorates during the IVD licensing process exemplified by the work of the WHO inspectorate during the process of Prequalification of IVDs. It also gave insights into the areas where non‐compliance deficiencies are most commonly found during inspections.

Dr. Nübling concluded the session with a presentation on *Risk Management* for IVD manufacturers. Starting from a standardised definition of risk according to ISO EN 14971, whereby risk is the probability of occurrence of harm combined with its severity, the manufacturer has to deal systematically with the risk of the individual IVD and provide this risk management as part of the quality management system as laid down in ISO 13485. This talk detailed the different steps of the risk management process, and explained the Failure Mode and Effects Analysis as one possible tool of risk management. The day ended with a question and answer session, allowing the participants to clarify some outstanding issues.

### 3.4 Day 3—Assessment of the IVD technical file: Part 2

This day started with *Product Performance Evaluations* and associated validation and verification studies for an IVD product. Two kinds of product performance studies are needed for the characterisation if IVDs, the analytical performance studies, which evaluate the ability of an IVD to detect or measure a particular analyte, that is a biomarker of interest, like a viral antigen; and the clinical performance studies, presented in the first talk by Prof. Rosanna Peeling (London School of Hygiene and Tropical Medicine) in the talk on evaluating clinical performances of diagnostics for blood screening. The presentation explained the relevance of donor screening to ensure a safe blood supply and then laid out how to evaluate the clinical performance of diagnostics for blood screening. *Clinical Performance Evaluation* is defined as a set of ongoing activities that use scientifically sound methods for the assessment and analysis of clinical data to verify the safety, clinical performance and/or effectiveness of the medical device when used as intended by the manufacturer.^9^ The focus here was on the aspects of reference standards and blinding, the source of clinical samples, the sample size necessary to evaluate the performance, and data management and analysis as important building block for a good evaluation of the clinical performance.

The talk of Dr Heiner Scheiblauer (IVD Testing Laboratory at PEI) elaborated on *Analytical Performance Evaluation*, which analyses the ability of an IVD to detect or measure a particular analyte, that is a biomarker of interest, like a viral antigen. The analytical data are generated using well‐characterised samples, for which the expected outcome is already known. This is in contrast to the aforementioned clinical performance studies that focus on the performance on clinical samples from patients for which the diagnostic tool is intended. By using these highly predictable samples, parameters like specimen type suitability, accuracy, trueness, traceability, precision, analytical sensitivity (limit of detection), analytical specificity (interferences and cross‐reactivity), assay measuring range, validation of assay cut‐off, robustness and high‐dose hook effect can be studied to characterise the performance of the assay. All the parameters were presented in detail and their relevance for the evaluation of IVDs was explained.

Mr. Washington Samukange proceeded with Stability and Robustness studies for blood screening diagnostics. This talk focused on the review of stability data for IVD products, regulatory requirements for stability, basic elements for stability testing, as well as sample stability. Participants got an opportunity to see the key points to look out for when reviewing stability data, and what to look out for. More importantly, they got a tour of the reference documents they can use as part of their review process. The session was drawn to a close with a talk given by Mr. Chancelar Kafere of the BloodTrain on *Labelling and Instructions for Use* (IFU) requirements. It is important to point out here that medical device labelling comprises not only the label on the device or its box, but also the Instructions For Use, the Information intended for the User and/or Patient, and all other information that come with the device.^10^ This presentation educated participants on the elements of medical device labelling, the available standards guiding labelling requirements and some general requirements for labels and the IFU. These requirements include, for example, the location of the information, the format and the required information on the label, as well as in the IFU. In general, the information provided should be readily understood by the intended user (professional or lay person) and be sufficiently comprehensive to allow the user a clear understanding, for what the IVD is intended, how long it can be used, how it is to be stored and so on. The presentation ended with a summary of common labelling non‐conformances to provide the participants with some practical examples, for example an incomplete claim for the intended use or the exclusion of limitations associated with the IVD.

### 3.5 Day 4—Global regulatory harmonisation for IVDs and WHO tools to facilitate access to IVDs

On this day the first talk from Ms. Anita Sands ended the series on the subheadings of the technical file by presenting *Post‐Market Surveillance* of blood screening IVDs. This was based on the WHO's guidance document for post‐market surveillance and market surveillance of medical devices including IVDs.^11^ The concept of planned Post‐Market Surveillance allows the manufacturer of an IVD to learn about problems on the market in order to ameliorate the product, so that all potential problems with the product can be sufficiently addressed in a timely manner. Post‐Marketing surveillance concepts always include information of the regulatory authorities to allow a re‐evaluation of the benefit/risk analysis of the IVD in cases of for example, malfunctions or unforeseen side‐effects. The presentation also detailed the roles of the user, the manufacturer and the regulator in the process of Post‐Marketing surveillance and discussed the relevance of lot testing of IVDs on the market.

Subsequently, several presentations were made by colleagues from the WHO to edify participants on several IVD‐related activities on‐going at the WHO. Dr Ana Aceves Capri introduced participants to the WHO Model List of Essential IVDs—a policy document based on scientific evidence, consisting of a list of categories of essential IVD tests and recommended assay formats for those tests. The goal is to provide evidence‐based guidance to countries for creating or updating their national essential IVD list and offer a reference to countries for prioritising the IVDs that should be available at different levels of the health‐care system. Ms. Marie Valentin presented the WHO Good Reliance Practices (GRelP). This presentation informed on the principles of GRelP and buttressed the importance of international cooperation to ensure the safety, quality and efficacy/performance of locally used medical products. Reliance makes best use of available resources and expertise, avoiding duplication and concentrating regulatory efforts and resources where it is most needed.

Dr Ute Ströher talked about the WHO Emergency Use Listing (EUL) procedure, citing examples of IVD for Ebola, Zika and SARS‐CoV‐2. The EUL procedure was set up as an extraordinary process intended to provide guidance to interested United Nations procurement agencies and NRAs on the quality, safety and performance of IVDs. It is a risk‐based approach to expedite the availability of IVDs needed in public health emergency situations. The EUL IVD assessment procedure was outlined in this presentation and includes assessment of a technical documentation relating to safety and performance (product information, product performance specifications and labelling), a review of manufacturing documentation and an on‐site inspection based on ISO 13485, and finally an independent laboratory evaluation coordinated by the WHO. Ms. Agnes Kijo concluded the session with a presentation on the facilitated regulatory pathways for medical products and the WHO Collaborative Procedure (CRP). This talk emphasised on the need for globalisation in the regulation of medical products, facilitated pathways to ‘transfer’ regulatory information and knowledge from trusted sources to facilitate in‐country approval, and how exactly the CRPs work. The example and lessons learned from a CRP that was used as a pilot to facilitate registration of IVDs in four NRAs (Ethiopia, Nigeria, Tanzania Mainland and Ghana) was presented. Following a very informative plenary discussion, Dr. Jens Reinhardt ended the workshop with closing remarks and a vote of thanks.

## 4 WORKSHOP OUTCOMES AND OUTLOOK/CONCLUSION

The participants of the virtual workshop obtained a systematic training on the composition and principles for assessing IVD technical files for blood screening IVDs, following the structure of the dossier. This information can be easily adapted and expanded to include further IVDs with intended uses being not for blood screening. Participants also grasped current information on the general regulatory requirements for IVDs. An awareness on the best practices in IVD regulation was created. In addition, an overview of the current situation of IVD regulation in four countries was provided to allow participants to profit from the experiences gathered by the countries and the challenges facing the establishment of effective IVD regulation. Participants also took home a vivid understanding of how to implement reliance models for their specific country situations. The concept of EUL or authorization for diagnostics and other health products was also introduced to participating NRAs to better prepare them for emergencies. We strongly believe that all vital information obtained in this workshop will help to enable countries implanting a good foundation as they begin the basics of IVD regulation. In 2022, BloodTrain is planning several face‐to‐face on‐site meetings with the individual partner NRAs in Africa, to instil the practicalities and technical skills involved with assessing dossiers by also including detailed training with case studies. This will help the assessors to be more effective in critically reviewing and assessing IVD technical files submitted to their respective jurisdictions. Feedback obtained from the workshop participants was overall very positive, with many of them testifying to the relevance and timeliness of such a workshop, and looking forward to more workshops on IVDs.

The online workshop rounded off smoothly with almost no technical hitches thanks to the e‐learning platform of the PEI‐GHPP projects through which the workshop was hosted.

## CONFLICT OF INTEREST

The authors declare no conflict of interest.
